# Übersterblichkeit von COVID-19-negativen Patienten mit proximaler Femurfraktur während der Pandemie: Was können wir für zukünftige Pandemien lernen?

**DOI:** 10.1007/s00113-025-01572-z

**Published:** 2025-05-14

**Authors:** Jakob Mayr, Anna Kurnoth, Nora Koenemann, Timon Röttinger, Leonhard Lisitano, Edgar Mayr, Annabel Fenwick

**Affiliations:** 1 Klinik für Unfallchirurgie, Orthopädie, Plastische- und Handchirurgie, Universitätsklinikum Augsburg, Stenglinstraße 2, 86156 Augsburg, Deutschland; 2 Zentrum für Unfallchirurgie und Orthopädie, Klinikum Ingolstadt GmbH, Krumenauerstraße 25, 85049 Ingolstadt, Deutschland

**Keywords:** Komplikationen, Fragilitätsfraktur, Soziale Distanzierung, Mortalität, Orthogeriatrisches Komanagement, Complications, Fragility fracture, Social distancing, Mortality, Orthogeriatric co-management

## Abstract

**Hintergrund:**

Die globale COVID-19-Pandemie führte zu einer hohen Übersterblichkeit, insbesondere bei vulnerablen älteren Patienten mit simultanen Begleiterkrankungen. Patienten mit proximalen Femurfrakturen weisen bereits ein sehr hohes Sterblichkeitsrisiko bis zu 30 % im ersten postoperativen Jahr auf. Ziel dieser Studie ist es, nicht nur die Auswirkungen von COVID-19 auf die Mortalität bei positiv-getesteten Patienten, sondern auch bei negativ-getesteten Patienten mit proximalen Femurfrakturen zu untersuchen.

**Methoden:**

Es wurde eine retrospektive Single-center-Kohortenstudie mit 2186 Patienten, die aufgrund einer proximalen Femurfraktur operativ behandelt wurden, an einem überregionalen Traumazentrum durchgeführt. Dabei wurden die Sterblichkeits- und die Komplikationsrate vor der COVID-19-Pandemie (Januar 2016 bis Februar 2020) und während der Pandemie (März 2020 bis Oktober 2021) verglichen. Es wurde während des gesamten Beobachtungszeitraums ein einheitliches Behandlungsprotokoll durchgeführt. Das orthogeriatrische Komanagement wurde durch die Pandemie negativ beeinträchtigt. Ausgewertet wurden Patientendaten, SARS-CoV-2-Infektion, chirurgisches Verfahren, Zeit bis zur Operation, Komplikationen und Sterblichkeit.

**Ergebnisse:**

Die Pandemiegruppe umfasste 596 Patienten mit einem Durchschnittsalter von 79,7 Jahren. Während der Pandemie wurden 26 Patienten (18 Frauen, 8 Männer, Durchschnittsalter 81,4 Jahre; Min: 63 Jahre, Max: 99 Jahre; SD ± 9 Jahre) positiv auf COVID-19 getestet. Patienten, die positiv auf COVID-19 getestet wurden, hatten im gleichen Zeitraum deutlich mehr Komorbiditäten als die COVID-19-negativen Patienten (CCI: 6,26 vs. 5,25 Punkte, *p* < 0,037). Die Kontrollgruppe vor der Pandemie bestand aus 1590 Patienten mit einem Durchschnittsalter von 79,9 Jahren und einem mittleren CCI von 5,86 Punkten. Positiv-getestete Patienten hatten einen deutlich längeren Krankenhausaufenthalt, eine längere Verweildauer auf der Intensivstation (*p* < 0,001) und eine Komplikationsrate von 62,5 %, insbesondere bezüglich des Auftretens einer Lungenentzündung (*p* < 0,001). Die Sterblichkeitsrate während der Pandemie unterschied sich nicht zwischen positiv-getesteten und negativ-getesteten Patienten, war jedoch bei beiden Gruppen im Vergleich zur Zeit vor der Pandemie signifikant höher (Pandemie: 14 % vs. 15,4 %, vor der Pandemie: 3,1 %).

**Schlussfolgerung:**

Patienten mit einer proximalen Femurfraktur und einer SARS-CoV-2-Infektion haben ein hohes Risiko für Komplikationen und eine hohe Sterblichkeit. Der Gesamtanstieg der Sterblichkeit aller Patienten mit kritischen Verletzungen wie Hüftfrakturen während der Pandemie unterstreicht die Bedeutung einer frühzeitigen Mobilisierung und eines orthogeriatrischen Komanagements, das während der Pandemie und des Lockdowns ausgesetzt wurde.

**Graphic abstract:**

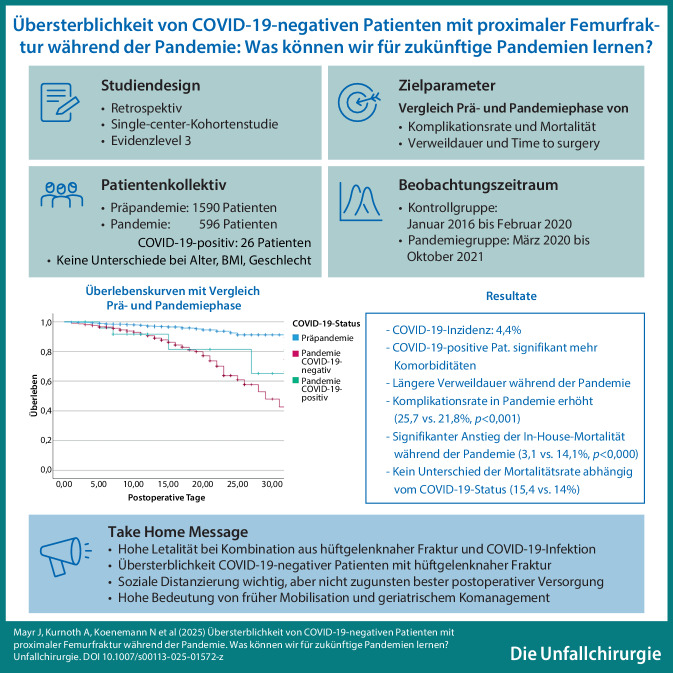

## Abstract (en)

**Background:**

The global COVID-19 pandemic led to excess mortality, especially for vulnerable older patients with simultaneous comorbidities. Patients with proximal femoral fractures already have a high mortality risk of up to 30% during the first postoperative year. The purpose of this study was not only to investigate the impact of COVID-19 on the mortality of patients tested positive but also of negatively tested patients with proximal femoral fractures.

**Methods:**

A single center cohort study of 2186 patients (mean age 79.8 years) who were surgically treated for a proximal femoral fracture at a level I trauma center was retrospectively carried out. The mortality and complication rates before the COVID-19 pandemic (January 2016–February 2020) and during the pandemic (March 2020–October 2021) were compared. A standardized treatment protocol was carried out during the total observational period. Orthogeriatric co-management was negatively impaired by the pandemic. Patient data, COVID-19 infections, surgical procedure, time from admission to surgery, postoperative complications and mortality were analyzed.

**Results:**

The pandemic group included 596 patients with an average age of 79.7 years. During the pandemic 26 patients were tested positive for COVID-19 (18 women, 8 men, average age 81.4 years, minimum 63 years, maximum 99 years, SD 9 years). Patients who tested positive for COVID-19 had more comorbidities than COVID-19 negative patients during the same period (Charlson comorbidity index, CCI 6.26 vs.5.25 points* p* < 0.037). The prepandemic control group consisted of 1590 patients with an average age of 79.9 years and a mean CCI of 5.86 points. Patients tested positive had a much longer hospitalization and a longer duration on the intensive care unit (*p* < 0.001) and a complication rate of 62.5%, especially due to the occurrence of pneumonia (*p* < 0.001). The mortality rate during the pandemic did not differ between patients tested positive or patients tested negative but was significantly higher for both groups in comparison to the period before the pandemic (pandemic 14% vs. 15.4%, before the pandemic 3.1%).

**Conclusion:**

Patients suffering from COVID-19 infection and proximal femoral fractures have a high risk of complications and a high mortality. The total increase in mortality for all patients with critical injuries, such as hip fractures during the pandemic emphasizes the importance of early mobilization and orthogeriatric co-management, which was discontinued during the pandemic and the lockdown.

## Hintergrund und Fragestellung

Die weltweite COVID-19-Pandemie führte zu einer hohen Übersterblichkeit, insbesondere bei vulnerablen älteren Patienten mit entsprechenden Begleiterkrankungen [1]. Die Pandemie stellte eine massive Belastung für das ohnehin schon angeschlagene Gesundheitssystem dar und stellte die Krankenhäuser vor die große Aufgabe, eine große Anzahl infizierter und infektiöser Patienten und eine steigende Anzahl an Patienten, die einer intensivmedizinischen Betreuung bedurften, zu bewältigen. Dies führte weltweit zu begrenzten Kapazitäten in der Intensiv- und Akutmedizin sowie zu einem Mangel an medizinischem Personal [2]. Es ist erwiesen, dass Patienten mit proximalen Femurfrakturen ein sehr hohes Sterberisiko im Krankenhaus und ein Sterberisiko bis zu 30 % im ersten postoperativen Jahr haben [3]. Diese meist geriatrischen Patienten waren besonders anfällig für die Risiken der COVID-19-Pandemie [4]. Die chirurgische Behandlung von Hüftfrakturen stellt eine dringliche Indikation dar [5]. Die eingeführten Lockdown-Regelungen führten zu einem Rückgang der Patienten mit akuten Verletzungen, d. h. Frakturen bei jungen Menschen, z. B. Sprunggelenks- und Wirbelsäulenfrakturen nach körperlicher Aktivität oder Hochrasanztraumata [6]. Die bundesweite Frakturerfassung in Deutschland zeigte jedoch retrospektiv einen leichten Anstieg der proximalen Femurfrakturen und anderer Fragilitätsfrakturen bei Patienten über 65 Jahren [7]. Studien haben gezeigt, dass eine frühzeitige Operation innerhalb von 48 h nach der Einlieferung für das postoperative Ergebnis und die Verringerung von Komplikationen und Mortalität nach proximalen Femurfrakturen von Vorteil ist [8]. Geriatrische Patienten mit Hüftfrakturen benötigen häufig eine postoperative intensivmedizinische Behandlung [9, 10]. Die steigende Patientenzahl und die Notwendigkeit der Intensivpflege kollidierten mit den begrenzten Personal- und Ressourcenkapazitäten des Krankenhauses.

Diese Studie untersucht die Auswirkungen der COVID-19-Pandemie auf die Sterblichkeit in einem überregionalen Traumazentrum mit einer spezialisierten orthogeriatrischen Abteilung. Das Traumazentrum stellte zudem ein überregionales Zentrum für die Behandlung von COVID-19 infizierten Patienten mit einem großen Einzugsgebiet dar. Unsere Studie ist jedoch eine von wenigen, die die Auswirkungen der Pandemie selbst auf Patienten mit proximalen Femurfrakturen, die nicht positiv auf COVID-19 getestet wurden, untersucht. Was kann die Unfallchirurgie hieraus für zukünftige Pandemien lernen?

## Material und Methoden

### Datenerhebung

Für die durchgeführte retrospektive Single-center-Kohortenstudie, Evidenzstufe III, wurden alle Patienten ausgewertet, die zwischen Januar 2016 und Oktober 2021 in unserem überregionalen Traumazentrum wegen einer proximalen Femurfraktur (Schenkelhalsfraktur, per-/subtrochantäre Fraktur) operativ behandelt wurden. Die Patienten während der COVID-19-Pandemie (März 2020 bis Oktober 2021) wurden analysiert und mit der Kontrollgruppe vor der Pandemie und der Gruppe der COVID-19-negativen Patienten während der Pandemie verglichen. Ausschlusskriterien waren: primär konservative Behandlung, Frakturen des Trochanter major, periprothetische Frakturen sowie Überweisungen für Revisionsoperationen, polytraumatisierte Patienten mit einem Injury Severity Score (ISS) > 9 und fehlende Informationen zum COVID-19-Status. Die durchgeführte Studie wurde von der örtlichen Ethikkommission genehmigt und erfüllt die Standards der Erklärung von Helsinki (20-2155-101). Die Krankenblätter wurden auf demografische Daten wie Alter, Geschlecht, BMI, Komorbiditäten, einschließlich des Charlson Comorbidity Index (CCI) [11] und Klassifikation der American Society of Anesthesiologists (ASA) [12], sowie auf Frakturmorphologie, Medikation, insbesondere Antikoagulanzien, Revisionsoperationen und SARS-CoV-2-Infektionen überprüft. Wurden die Patienten innerhalb des oben genannten Zeitraums für die kontralaterale Seite erneut aufgenommen, wurden sie erneut als separater Fall berücksichtigt. Analysiert wurden Art und Zeitpunkt der Operation ab der Aufnahme, die Dauer des Aufenthalts auf der Intensivstation (ICU) sowie die Gesamtdauer des Krankenhausaufenthalts (LHS). Komplikationen wurden erfasst und unterteilt in Harnwegsinfektionen, Lungenentzündung, einschließlich COVID-19-Pneumonie, Embolie oder Thrombose, Hämatom, Wundinfektionen, mechanische Komplikationen, d. h. postoperative Fraktur, Luxation oder „cutting out“. Für beide Gruppen wurden die Sterblichkeitsrate und die Todesursache im Krankenhaus ausgewertet.

### Therapie

Für den gesamten untersuchten Zeitraum galt das identische Therapieprotokoll. Die Zielzeit bis zur Operation lag innerhalb von 24 h nach Aufnahme in der Notaufnahme für alle Patienten ohne Antikoagulation oder nur mit Thrombozytenaggregationshemmung (TAH), einschließlich der dualen TAH-Therapie. Patienten mit direkten Antikoagulanzien wurden entsprechend ihrer Nierenfunktion in 2 Gruppen eingeteilt (Gruppe 1: Glomeruläre Filtrationsrate [GFR] > 50, Gruppe 2: GFR:< 50). Bei entsprechend guter Nierenfunktion wurde die Operation innerhalb von 24 h durchgeführt. Bei eingeschränkter Nierenfunktion wurde die Operation auf 24 –48 h nach der Aufnahme verschoben, um das Blutungsrisiko zu verringern. Der Status der SARS-CoV-2-Infektion nahm keinen Einfluss auf das Therapie- und Zeitprotokoll. Je nach präoperativer Mobilität und Komorbiditäten sowie Frakturmorphologie wurden bei Schenkelhalsfrakturen eine Total- oder Hemiendoprothese (zementiert oder unzementiert; Fa. Zimmer Biomet, Indiana, USA), bei per-/subtrochantären Frakturen eine Marknagelung (Proximaler Femurnagel [PFNa]; Fa. Synthes, Oberdorf, Schweiz; ± Cerclage) und bei jungen Patienten mit Schenkelhalsfrakturen kopferhaltend eine Dynamische Hüftschraube DHS/Schraubenosteosynthese durchgeführt. Alle Patienten erhielten 30 Minuten vor Schnitt eine Einzeldosis von 2 g Cefazolin i.v.

Postoperativ wurde vom ersten Tag an eine venöse Thromboembolieprophylaxe mit Enoxaparin, 40 mg s.c., durchgeführt. Die Antikoagulanzien wurden je nach Gewicht des Patienten durch Innohep® (Tinzaparin) gewichtsadaptiert ersetzt. Alle Patienten durften unmittelbar nach der Operation voll belasten mit Ausnahme von jungen Patienten, die bei DH-Klinge eine Teilbelastung umsetzen können, und erhielten vom ersten Tag an Physiotherapie.

Bei Aufnahme erfolgt ein geriatrisches Screening. Folgende Kriterien werden dabei beachtet und Punkte mit unterschiedlicher Wichtung vergeben. Ab 4 Punkten wird ein Patient als geriatrisch eingestuft:

- Alter ab 70 Jahren,
- Gangunsicherheit/Gehhilfen/Rollator,
- Verwirrtheit/Demenz/Delir,
- starke Sehbehinderung,
- starke Hörminderung,
- Stürze (ab 2 im vergangenen Jahr),
- Multimedikation (ab 5 Medikamenten),
- Mangelernährung.

Alle geriatrisch definierten Patienten wurden auf einer orthogeriatrischen Station mit intensiver täglicher Physio- und Ergotherapie sowie Gruppentherapie behandelt. Die Patienten erhielten mindestens 20 Therapieeinheiten (Logo, Ergo- und Physiotherapie) innerhalb von 14 Tagen, von denen nur 2 Gruppentherapien seien durften. Zusätzlich fanden tägliche Gruppentherapien statt. Es erfolgten eine tägliche interdisziplinäre Visite sowie eine Teambesprechung und Kurvenvisite. Die tägliche Beschäftigungstherapie (u. a. Musizieren, Quizrunden, Töpfern und Handwerken, Singen) fand in einem Gemeinschaftsraum, in dem die Patienten auch ihre Mahlzeiten einnahmen, statt, um eine klassische „Patientenkarriere“ zu vermeiden und ein selbstbestimmtes Leben zu erhalten. Ausschlusskriterien waren fortgeschrittene Demenz oder präoperative Immobilität.

Die Patienten wurden als positiv eingestuft, wenn der PCR-Test positiv für eine SARS-CoV-2-Infektion war. Alle Patienten wurden bei der Aufnahme in der Notaufnahme und wöchentlich auf der Station sowie verpflichtend vor der Entlassung mittels PCR getestet. Bei einem akuten Ausbruchsgeschehen wurde alle 2 Tage getestet. War der Test zu irgendeinem Zeitpunkt positiv, wurden die Patienten umgehend isoliert. Die Isolierung wurde in allen Bereichen des Krankenhauses strikt eingehalten. In der Notaufnahme wurden getrennte Bereiche geschaffen, separate COVID-19-Stationen mit reduziertem Personalwechsel und COVID-19-Intensivstationen wurden gemäß den allgemeinen Hygienerichtlinien des Krankenhauses eingerichtet. Ein OP, der sich in einiger Entfernung von den anderen OP befand und über separate Waschräume, einen direkten Zugang zu Umkleideräumen und eine von der Intensivstation getrennte Patientenaufnahme verfügte, wurde für positive Patienten aller Fachrichtungen genutzt. Das orthogeriatrische Management war während der Pandemie aufgrund von Hygieneproblemen stark beeinträchtigt bis vollständig ausgesetzt. Der Gemeinschaftsraum durfte nicht benutzt werden. Therapieeinheiten waren aufgrund von personellen Engpässen drastisch reduziert, und Gruppentherapie durfte nicht stattfinden. Tägliche Visiten und Kurvenvisiten wurden weiterhin durchgeführt. COVID-19-positive Patienten erhielten kein orthogeriatrisches Management, da diese Patienten auf getrennten Stationen behandelt wurden. Die COVID-19-Stationen wurden von ärztlichem Personal unterschiedlicher Fachrichtungen betreut.

### Statistik

Die statistische Analyse wurde mit IBM SPSS Statistics (Version 27; IBM Deutschland Ltd., Ehningen, Deutschland) durchgeführt. Die Normalverteilung aller Daten wurde überprüft. Der Student’s *t*-Test, und Chi-Quadrat wurden verwendet, um Unterschiede und Einflussfaktoren in Bezug auf Komplikationen und Mortalität zu ermitteln; 95 %-Konfidenzintervalle und Standardabweichungen wurden berechnet. Für Daten ohne Normalverteilung wurde der Wilcoxon-Rang-Test verwendet. Für die Beschreibung signifikanter Unterschiede in der Sterblichkeit zwischen den Gruppen wurde der Exakte Test von Fisher verwendet. Die Überlebensanalyse wurde mit Kaplan-Meier-Kurven dargestellt. Das Signifikanzniveau wurde auf 5 % (α = 0,05) festgelegt.

## Ergebnisse

### Demografische Daten

Die Pandemiegruppe umfasste 596 Patienten, die überwiegend weiblich waren (67,4 % weiblich und 32,6 % männlich). Das Durchschnittsalter betrug 79,69 Jahre (Spanne: 24 Jahre bis 99 Jahre; SD 12,3). Der mittlere BMI betrug 24 kg/m^2^ (Spanne: 11,7–66 kg/m^2^). Die Kohorte umfasste 258 Schenkelhalsfrakturen (43,5 %) und 327 pertrochantäre Frakturen (56,5 %). In 115 Fällen wurde eine Totalendoprothese (TEP) implantiert. Weitere 115 Patienten erhielten eine Hemiendoprothese (HEP). Eine Marknagelung wurde in 327 Fällen durchgeführt, 27 Patienten erhielten eine dynamische Hüftschraube (DHS) und ein Patient eine Schraubenosteosynthese.

Die Kontrollgruppe vor der Pandemie bestand aus 1590 Patienten mit einem Durchschnittsalter von 79,9 Jahren (Spanne: 23 Jahre bis 103 Jahre, SD: 11,7) und war ebenfalls überwiegend weiblich (69,1 %). Die Verteilung der Frakturtypen zeigte 769 Schenkelhalsfrakturen (48,4 %), 821 per-/subtrochantäre Frakturen (51,6 %), die mit 342 TEP, 393 HEP und 816 Fällen von intramedullärer Nagelosteosynthese und 39 Fällen von Osteosynthese behandelt wurden. Zwischen den Gruppen gab es keine Unterschiede in Bezug auf BMI, Alter oder Geschlechterverteilung.

### Präoperativer Status

**Abb. 1:**
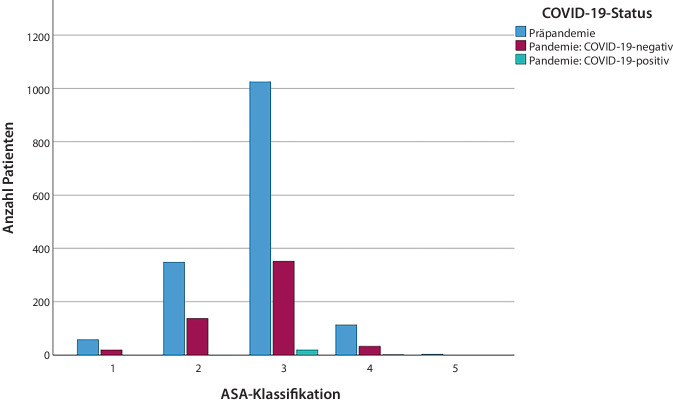
Verteilung der ASA-Klassifikation präpandemisch und während der Pandemie (COVID-19-positiv und -negativ)

In der Kontrollgruppe lebten 963 Patienten (60,6 %) ohne Betreuung selbstversorgt, während 39,4 % bereits vor der Krankenhausaufnahme betreut wurden (Pflegegrade: PG 1: 121, PG 2: 229, PG 3: 152; PG 4: 111, PG 5: 9). In der Pandemiegruppe lebten 279 Patienten (46,9 %) ohne Betreuung selbstständig, während 53,1 % bereits vor der Krankenhauseinweisung betreut wurden (Pflegegrade: PG 1: 30, PG 2: 120, PG 3: 97; PG 4: 57, PG 5: 12). Die präoperative Mobilität war bereits bei 53,8 % der kompletten Studienkohorte eingeschränkt. Der durchschnittliche CCI in der gesamten Pandemiegruppe betrug 5,29 Punkte (Spanne: 0 Punkte bis 15 Punkte; SD 2455). Der mittlere CCI war in der präpandemischen Gruppe mit 5,86 Punkten etwas höher (Spanne: 0 Punkte bis 15 Punkte; SD 2,4). Die ASA-Klassifizierung für beide Gruppen ist in Abb. 1 dargestellt.

### COVID-19-positive Gruppe

Die Rate an positiv-getesteten Patienten mit einer proximalen Femurfraktur lag im Untersuchungszeitraum bei 4,4 %. Während der Pandemie wurden 26 Patienten (18 Frauen, 8 Männer, Durchschnittsalter 81,35 Jahre; SD 9 Jahre) positiv auf COVID-19 getestet. Alle Antigen Schnelltests wurden durch PCR-Tests bestätigt. In der Gruppe der positiv-getesteten Patienten waren 10 Schenkelhalsfrakturen und 16 pertrochantäre Frakturen. Patienten, die positiv auf COVID-19 getestet wurden, wiesen im gleichen Zeitraum signifikant mehr Komorbiditäten auf als die negativen Patienten (CCI: 6,26 vs. 5,25 Punkte; *p* < 0,037), (Tab. 1).

**Tab. 1:** Demografische Daten und Mortalität im Vergleich zwischen Präpandemie und Pandemie

Untersuchte Variable	Präpandemie	Pandemie		*p*-Wert
		*COVID-19-positiv*	*COVID-19-negativ*	
Anzahl der Patienten	1590	26	570	–
Alter	79,9	81,35	79,8	< 0,05 (Zwischen Prä – und Gesamtpandemie)
Komplikationsrate in %	21,8	62,5	25,7	0,001 (Zwischen prä – und Gesamtpandemie sowie zwischen COVID-19-positiv und -negativ)
Verweildauer In Tagen	13,5	16,7	14,04	0,001 (Zwischen Prä – und Gesamtpandemie)
CCI in Punkten	5,86	6,26	5,25	0,037 (Zwischen Prä – und Gesamtpandemie sowie zwischen COVID-19-positiv und -negativ)
Mortalitätsrate in %	3,1 (*n* = 50)	15,4 (*n* = 4)	14 (*n* = 78)	0,001 (Kein Unterschied in den Pandemiegruppen)

### Zeit bis zur Operation

Im Durchschnitt wurde die Operation während der Pandemie 23,2 h (SD 19,1 h) nach der Krankenhausaufnahme durchgeführt. Die Wartezeit für positiv-getestete Patienten war kürzer, aber nicht signifikant (22,87 h vs. 23,26 h; *p* < 0,7). In der Kontrollgruppe betrug die durchschnittliche Wartezeit 26,4 h (Bereich: 0,95–140 h; SD 19,6 h; *p* < 0,001).

### Verweildauer

In der Kontrollgruppe betrug die mittlere Aufenthaltsdauer 13,5 Tage (SD 6,8 Tage). Im Durchschnitt wurden die Patienten nach der Operation 0,48 Tage lang auf der Intensivstation behandelt (Bereich: 0 Tage bis 22 Tage). Während der Pandemie blieben COVID-19-negative Patienten 14,04 Tage (SD: 6,5 Tage) und COVID-19-positive Patienten 16,7 Tage (SD: 10,4 Tage) im Krankenhaus. Der postoperative Aufenthalt auf der Intensivstation war bei positiv-getesteten Patienten deutlich länger (2,2 Tage gegenüber 0,31 Tagen; *p* < 0,001).

### Komplikationen und Mortalität

Während der Pandemie lag die Gesamtkomplikationsrate bei 25,7 %. Harnwegsinfektionen traten bei 60 Patienten (10,1 %) auf und wurden mit Antibiotika behandelt. Eine Lungenentzündung wurde bei 33 (6 %) Patienten (Röntgen, Labor und Symptome) mit negativem Test und bei 45,8 % der positiv auf COVID-19 getesteten Patienten festgestellt und ebenfalls mit Antibiotika i.v. behandelt. In der kleinen Gruppe der positiven Patienten traten in 62,5 % der Fälle Komplikationen auf (*p* < 0,001). Nur ein positiver Patient hatte eine Harnwegsinfektion. In 9 Fällen trat eine postoperative Wundinfektion auf (1,5 %). Der häufigste Erreger war *Staphylococcus aureus*. In 2 Fällen kam es zu rezidivierenden Luxationen mit Wechsel auf eine tripolare Pfanne, in 2 Fällen zu einer periprothetischen Fraktur. Im Rahmen vom Osteosyntheseversagen bzw. Cutting out erfolgt der Wechsel auf eine Endoprothese. Eine Wunddehiszenz nach Manipulation durch die Patientin musste revidiert werden.

Die Gesamtkomplikationsrate vor der Pandemie war mit 21,8 % niedriger (*p* < 0,001). Bei den chirurgischen Komplikationen handelte es sich um Infektionen (*n* = 20), die eine Revisionsoperation und eine i.v.-Antibiotikagabe erforderlich machten, sowie um periimplantäre oder prothetische Frakturen, Cutting out und Luxationen nach Endoprothese (insgesamt *n* = 61). Im Fall einer Infektion war der häufigste Erreger *Staphylococcus aureus*. Harnwegsinfektionen traten bei 161 Patienten (10,1 %) auf und wurden mit Antibiotika behandelt. Eine Lungenentzündung wurde bei 87 (5,5 %) Patienten festgestellt (Röntgenbild, Laborwerte und Symptome) und ebenfalls mit Antibiotika i.v. und Mobilisierung behandelt. Es gab keinen Unterschied in der Komplikationsrate zwischen den angewendeten chirurgischen Verfahren. Die Dauer des Krankenhausaufenthalts war im Fall von Komplikationen wesentlich länger (18,2 vs. 12,2 Tage; *p* < 0,001). Die Gründe für die häufigste Sterblichkeit im Krankenhaus während der Pandemie waren Lungenembolie (*n* = 6), Herzstillstand und kardiogene Ursachen (*n* = 14), respiratorische Insuffizienz (*n* = 10), Pneumonie, einschließlich Aspiration und SARS-CoV-2-Infektion mit ARDS (*n* = 14), und Sepsis ± Multiorganversagen (*n* = 24). Die weiteren Todesursachen waren GI-Blutungen (*n* = 2) und Einstellen der Therapie und palliatives Setting bei fortgeschrittenem Tumorleiden (*n* = 10). In 2 Fällen war keine klare Todesursache zu eruieren.

Die hausinterne Sterblichkeitsrate vor der Pandemie betrug 3,1 % (*n* = 50). Die Todesursachen waren: Pneumonie (*n* = 12), Sepsis, ggf. mit Multiorganversagen (*n* = 13), Lungenarterienembolie (*n* = 4), intraoperative Zementembolie (*n* = 2), kardiale Ursache (*n* = 8), Hohlorganperforation (*n* = 2), respiratorische Insuffizienz (*n* = 4), Aspiration (*n* = 4) und GI-Blutung (*n* = 1).

**Abb. 2:**
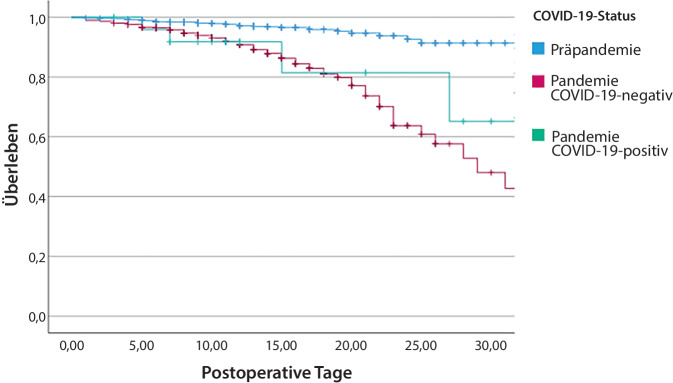
Kaplan-Meier-Überlebensrate, Präpandemie und Pandemieperiode im Vergleich (30-Tage-Mortalität)

Die Gesamtmortalitätsrate in der Pandemiegruppe lag bei 14,1 % (*p* < 0,001). 78 Patienten starben ohne einen positiven Coronatest und 4 in der COVID-19-Gruppe (14 % vs. 15,4 %, *p* < 0,8; Tab. 1). Die Sterblichkeitsrate während der Pandemie unterschied sich nicht zwischen positiv- und negativ-getesteten Patienten, war aber bei beiden Gruppen im Vergleich zur Zeit vor der Pandemie deutlich höher (Abb. 2).

## Diskussion

Proximale Femurfrakturen haben massive Auswirkungen auf das tägliche Leben und insbesondere auf die postoperative Mortalität [13, 14]. Die COVID-19-Pandemie führte zu einer überhöhten Sterblichkeit, die v. a. ältere Patienten mit Komorbiditäten betraf; diese bilden auch die Gruppe mit dem größten Risiko für proximale Femurfrakturen [1]. Alter, chronische Krankheiten (Herz-Kreislauf- und Atemwegserkrankungen), Diabetes mellitus, Ethnie und männliches Geschlecht, Fettleibigkeit und die Prävalenz von Krebserkrankungen wurden als Hauptfaktoren, die den Tod im Zusammenhang mit der SARS-CoV-2-Infektion beeinflussen, ermittelt [15, 16]. Während die Frakturrate bei jungen Menschen zurückging, konnten Studien zeigen, dass die Zahl der proximalen Femurfrakturen während der Pandemie und sogar während des Lockdowns zunahm [6, 7]. Die von uns gemeldete Inzidenz von 4 % der Patienten mit proximalen Femurfrakturen und SARS-CoV-2-Infektion deckt sich mit bereits veröffentlichten Studien [17–19]. In einer Metaanalyse wurden Inzidenzen bis zu 20 % angegeben [20]. Während die Gesamtzahl der orthopädischen/traumatologischen Operationen während der Pandemie zurückging, zeigte sich bei den Fragilitätsfrakturen kein signifikanter Rückgang [21]. Daten aus der US-amerikanischen Premier Healthcare Database zeigten einen Anstieg der Sterblichkeit von Patienten mit proximalen Femurfrakturen und einer gleichzeitigen SARS-CoV-2-Infektion (OR 2,85) im Gegensatz zu Patienten, die aufgrund einer proximalen Femurfraktur ohne SARS-CoV-2-Infektion operiert wurden [22]. In unserer Kohorte fanden wir nur einen geringen Anstieg der Sterblichkeitsrate bei Patienten, die sowohl eine proximale Femurfraktur als auch eine SARS-CoV-2-Infektion aufwiesen, im Gegensatz zu den COVID-19-negativen Patienten mit einer proximalen Femurfraktur während der Pandemie. Die hausinterne Sterblichkeitsrate von 3,1 % vor der Pandemie ist niedrig. Wir konnten jedoch nachweisen, dass die Gesamtsterblichkeitsrate während der Pandemie im Vergleich zur Zeit vor der Pandemie erheblich anstieg.

Was könnte zu diesem Anstieg geführt haben? Die soziale Distanzierung wurde auch im Krankenhaus strikt eingehalten. Auf unserer orthogeriatrischen Station konnten Gruppentherapien und Gruppentreffen sowie soziale Aktivitäten nicht stattfinden. Dies könnte zu einer geringeren Mobilisierung und einer stärkeren sozialen Isolation und Abnahme der Motivation der Pandemiegruppe geführt haben [19].

Im Gegensatz zu unseren Ergebnissen haben Studien sogar eine niedrigere Sterblichkeitsrate und eine kürzere Verweildauer auf der Intensivstation bei COVID-19-positiven Patienten nachgewiesen und folgerten hieraus, dass dies auf die kürzere Zeit bis zur Operation und die frühzeitige Entlassung der Patienten zurückzuführen ist [23]. Lachnish et al. [24] kamen zu dem Schluss, dass positiv-getestete Patienten von einer frühen Operation und Entlassung profitiert haben. Wir berichteten auch über kürzere Zeiten von der Aufnahme bis zur Operation, was jedoch in unserem Kollektiv nicht zu einer niedrigeren Sterblichkeitsrate führte. Wignall et al. [18] sahen während der Pandemie sogar einen Anstieg der nichtoperativen Behandlung proximaler Femurfrakturen. Dies kann durch unsere Ergebnisse nicht gestützt werden, da eine konservative Therapie von proximalen Femurfrakturen lediglich in Ausnahmesituationen z. B. bei palliativer Versorgung durchgeführt werden sollte und mit einer hohen Sterblichkeitsrate verbunden ist.

Eine groß angelegte Registerstudie mit über 15.000 Patienten, die das Jahr vor der Pandemie mit 2020 verglichen haben, zeigten ebenfalls signifikant kürzere Zeiten bis zur Operation ohne veränderte Inzidenz der hüftgelenknahen Frakturen und führten dies auf die Reduktion elektiver Eingriffe zurück. Interessanterweise zeigten sich keine erheblichen Unterschiede in der Mortalitätsrate vor und nach der Pandemie. Bei näherer Betrachtung von Zeiten hoher Inzidenz (> 50/100000) kamen die Autoren jedoch zu dem gleichen Ergebnis, dass die Sterblichkeit aller Patienten unabhängig vom SARS-CoV-2-Infektionsstatus signifikant anstieg [25].

Eine große Metaanalyse von Freitas et al. [26] kam zu dem Schluss, dass eine SARS-CoV-2-Infektion zum gleichen Zeitpunkt wie eine Operation bei proximalen Femurfrakturen ein 3,65fach höheres Sterberisiko darstellt und zu einem schlechteren klinischen Ergebnis führt. Studien zeigten 30-Tage-Mortalität-Raten bis zu 30 % nach Hüftfrakturen bei infizierten Patienten [27]. Anusitviwat et al. zeigten höhere Komplikationsraten von 36 % gegenüber 22 % während der Pandemie im Gegensatz zu Komplikationsraten vor der Pandemie und auch niedrigere funktionelle Ergebnisse im Barthel-Index dieser Patienten [28]. Diese Ergebnisse stimmen mit denen von Faggiani et al. [29] überein, die zu dem Schluss kamen, dass eine SARS-CoV-2-Infektion bei Patienten mit proximalen Femurfrakturen zu schlechteren radiologischen und funktionellen Ergebnissen und einer erhöhten Gesamtmortalität führte.

Verlegungen zwischen Krankenhäusern, wie sie während der Pandemie häufig vorkamen, führten ebenfalls zu einer höheren Sterblichkeitsrate. Vor Beginn der Impfung des medizinischen Personals waren die Infektionsraten im Krankenhaus erheblich höher. Die Impfung gegen COVID-19 zeigte auch eine Schutzfunktion nicht nur für das medizinische Personal, sondern auch für Patienten mit proximalen Femurfrakturen, da der Bedarf an postoperativer Intensivpflege sowie die Rate an postoperativen leichten Komplikationen reduziert wurden [30].

### Limitationen

Die Einschränkungen dieser Studie sind das retrospektive Design und das Fehlen von Langzeitdaten, wie z. B. einer Einjahresmortalitätsrate. Außerdem sind die kleine COVID-19-Kohorte zu berücksichtigen und die uneinheitliche Therapie aufgrund der zugrunde liegenden Veränderungen und Erfahrungen während der Pandemie. Es konnte keine Aussage zum Impfstatus der Patienten erhoben werden, wobei für den Erhebungszeitraum des ersten Jahres unserer Pandemiekohorte für die Allgemeinbevölkerung keine Impfung vorhanden war. Zudem wurde in der Studie nicht zwischen Zeiten hoher und niedriger Inzidenz unterschieden wie bei Knauf et al. [25]. Im Gegensatz zu veröffentlichten Daten zu diesem Thema präsentieren wir jedoch Ergebnisse mit einer beträchtlichen Anzahl von Patienten aus einem einzigen Traumazentrum und sind in der Lage, einen Vergleich mit einer großen Kontrollgruppe vor der Pandemie anzustellen.

## Ausblick

Die weltweite COVID-19-Pandemie führte zu einer hohen Sterblichkeitsrate, insbesondere bei geriatrischen Patienten mit Begleiterkrankungen. Bei Patienten, die sowohl an einer SARS-CoV-2-Infektion als auch an proximalen Femurfrakturen leiden, bestehen hohes Risiken für Komplikationen und eine hohe Sterblichkeit. Aber auch COVID-19-negative Patienten mit kritischen Verletzungen wie Hüftfrakturen sollten während einer Pandemie nicht vergessen werden, da sich auch bei ihnen ein erheblicher Anstieg der Sterblichkeit zeigte. Dies unterstreicht die Bedeutung der postoperativen Mobilisierung und Rehabilitation nach hüftgelenknahen Frakturen. Soziale Distanzierung ist wichtig, sollte uns aber nicht davon abhalten, die beste Behandlung zu ermöglichen, d. h. eine frühzeitige Mobilisierung und Physiotherapie für Patienten, die dies bei künftigen Pandemien dringend benötigen. Der Gesamtanstieg der Sterblichkeit bei Patienten mit kritischen Verletzungen wie Hüftfrakturen während der Pandemie unterstreicht zudem die Bedeutung einer frühzeitigen Mobilisierung und Aktivierung, z. B. im Rahmen des orthogeriatrischen Komanagements.

## Fazit für die Praxis

- Die Kombination aus SARS-CoV-2-Infektion und hüftgelenknahen Frakturen ist für geriatrische Patienten mit einer hohen Letalität verbunden.
- Die COVID-19-Pandemie führte auch zu einer Übersterblichkeit von COVID-19-negativen Patienten mit hüftgelenknahen Frakturen.
- Frühe Mobilisation und orthogeriatrisches Management von hüftgelenknahen Frakturen haben auch in Pandemiesituationen eine relevante Bedeutung.
- Eine soziale Distanzierung während einer Pandemie ist wichtig, sollte jedoch nicht auf Kosten einer optimalen postoperativen Therapie erfolgen.

## Data Availability

Die gesamte Datenbank der Studie ist verfügbar.
